# Compensatory changes in energy balance during dapagliflozin treatment in type 2 diabetes mellitus: a randomised double-blind, placebo-controlled, cross-over trial (ENERGIZE)—study protocol

**DOI:** 10.1136/bmjopen-2016-013539

**Published:** 2017-01-27

**Authors:** Surya Panicker Rajeev, Victoria S Sprung, Carl Roberts, Jo A Harrold, Jason C G Halford, Andrej Stancak, Emma J Boyland, Graham J Kemp, Daniel J Cuthbertson, John P H Wilding

**Affiliations:** 1 Institute of Ageing and Chronic Disease, University of Liverpool, Liverpool, UK; 2 Diabetes and Endocrinology Research Group, Clinical Sciences Centre, Aintree University Hospital NHS Foundation Trust, Liverpool, UK; 3 Department of Musculoskeletal Biology II, Institute of Ageing and Chronic Disease, University of Liverpool, Liverpool, UK; 4 Department of Psychological Sciences, Institute of Psychology, Health and Society, University of Liverpool, Liverpool, UK; 5 Magnetic Resonance and Image Analysis Research Centre (MARIARC), University of Liverpool, Liverpool, UK

## Abstract

**Introduction:**

Sodium glucose cotransporter 2 (SGLT2) inhibitors are effective blood-glucose-lowering medications with beneficial effects on body weight in patients with type 2 diabetes mellitus (T2DM). However, observed weight loss is less than that predicted from quantified glycosuria, suggesting a compensatory increase in energy intake or a decrease in energy expenditure. Studies using dual-energy X-ray absorptiometry (DEXA) have suggested most body weight change is due to loss of adipose tissue, but organ-specific changes in fat content (eg, liver, skeletal muscle) have not been determined. In this randomised, double-blind, placebo-controlled crossover study, we aim to study the compensatory changes in energy intake, eating behaviour and energy expenditure accompanying use of the SGLT2 inhibitor, dapagliflozin. Additionally, we aim to quantify changes in fat distribution using MRI, in liver fat using proton magnetic resonance spectroscopy (^1^H-MRS) and in central nervous system (CNS) responses to food images using blood oxygen level dependent (BOLD) functional MRI (fMRI).

**Methods and analysis:**

This outpatient study will evaluate the effect of dapagliflozin (10 mg), compared with placebo, on food intake and energy expenditure at 7 days and 12 weeks. 52 patients with T2DM will be randomised to dapagliflozin or placebo for short-term and long-term trial interventions in a within participants, crossover design. The primary outcome is the difference in energy intake during a test meal between dapagliflozin and placebo. Intake data are collected automatically using a customised programme operating a universal eating monitor (UEM). Secondary outcomes include (1) measures of appetite regulation including rate of eating, satiety quotient, appetite ratings (between and within meals), changes in CNS responses to food images measured using BOLD-fMRI, (2) measures of energy expenditure and (3) changes in body composition including changes in liver fat and abdominal visceral adipose tissue (VAT) and subcutaneous adipose tissue (SAT).

**Ethical approval:**

This study has been approved by the North West Liverpool Central Research Ethics Committee (14/NW/0340) and is conducted in accordance with the Declaration of Helsinki and the Good Clinical Practice (GCP).

**Trial registration number:**

ISRCTN14818531. EUDRACT number 2013-004264-60.

**Strengths and limitations of this study**

- This is the first study to address the discrepancy between the estimated and actual weight loss as a result of SGLT2 inhibition in a prospective within participant study design incorporating short-term and long-term effects.
- The study is designed to assess macrostructure and microstructure of eating behaviour and the use of imaging modalities provide a neurophysiological and mechanical correlate.
- The sample size is relatively small, although provides sufficient power to answer the primary research question.
- The measurement of energy expenditure is performed using indirect calorimetry as opposed to the gold standard whole body calorimetry.
- There may be a carryover effect on body weight and body composition changes since there is no wash-out period between the long-term cross over, but any effect of this on the primary outcome (food intake) should be minimal.

## Background and rationale

Type 2 diabetes mellitus (T2DM) is a disorder characterised by hyperglycaemia and insulin resistance, which leads to the development of microvascular and macrovascular complications. It is a major global public health challenge; the prevalence is expected to rise to 592 million by 2035.^1^ A range of pharmacological therapies to complement lifestyle interventions with diet and exercise are available including biguanides, sulphonylureas, thiazolidenediones, glucagon-like peptide-1 (GLP-1) agonists and dipeptidyl peptidase-IV (DPP-IV) inhibitors, as well as insulin. These therapies are usually unable to alter the natural progression of T2DM. Obesity is a major risk factor for T2DM, yet some treatments, particularly sulphonylureas, thiazolidenediones and insulin, may cause weight gain. Hence therapeutic agents that are weight neutral or associated with weight loss may be preferable. Inhibition of renal tubular reabsorption of glucose to induce glycosuria is effective in animal models^2^ and has led to the development of sodium/glucose cotransporter 2 (SGLT2) inhibitors for clinical use.

SGLT2 inhibitors such as dapagliflozin have been shown to cause significant reductions in glycaemia in patients with T2DM, comparable with those produced by other oral agents.^3–6^ In contrast to existing oral agents, treatment results in weight loss in all groups studied to date, including treatment-naive patients and those where SGLT2 inhibitors were added to other oral agents, insulin alone or a combination of oral agents with insulin. SGLT2 inhibition results in a net loss of ∼75 g glucose per day, equivalent to an energy loss of ∼300 kcal/day (1200 kJ/day), and also a diuresis (∼400 mL/day), resulting in a modest intravascular volume depletion (evidenced by a rise in haematocrit). The predicted weight loss over 24 weeks treatment with dapagliflozin (assuming no compensatory changes in either food intake or energy expenditure and no diuresis) based on the calculated caloric loss would be ∼7 kg. However, clinical data so far suggest that the observed total weight loss with dapagliflozin 10 mg dose is 2.5–3.2 kg (1.7–2.0 kg placebo-subtracted), that is, an actual (placebo-subtracted) energy deficit of ∼320 kJ/day, which is substantially less than the measured urinary energy losses.

This suggests that with chronic treatment there are compensatory mechanisms that attenuate weight loss.^7^ These could include either an increase in food intake, driven by hunger (weakened satiety) and increased responsiveness to food cues (reward driven wanting), or a reduction in energy expenditure. As in human T2DM, rats with dietary-induced obesity lose weight when treated with dapagliflozin (∼4%), but this is offset by a 30% compensatory increase in energy intake.^8^ Furthermore, pair-feeding matched to vehicle-treated animals leads to greater weight loss of ∼13%. In SGLT2 knockout mice, food intake was greater during the dark cycle than controls; physical activity also increased, energy expenditure was higher and respiratory quotient (RQ) fell, consistent with a shift from carbohydrate to fat metabolism. Human studies also demonstrate a shift from glucose to fat metabolism.^9^ Overall these data are consistent with the observations in humans and suggest that some compensatory increase in food intake occurs that limits the weight loss in dapagliflozin treatment; the metabolic changes also suggest that fuel usage may shift from carbohydrate to fat metabolism.^10^ Considering the associated weight loss, improved glycaemia and preference for fat metabolism, we believe that liver fat may also be reduced, an effect observed with GLP-1 receptor agonists.^11^

This study is designed to examine the behavioural and biological mechanisms underlying the changes in energy balance that occur with treatment with the SGLT2 inhibitor, dapagliflozin, to evaluate changes in body composition and also to help answer important physiological questions regarding adaptive responses to a state of negative energy balance mediated by promoting glycosuria.

## Primary objective

To evaluate the effect of dapagliflozin, 10 mg daily, compared with placebo, when added to up to two other oral glucose-lowering medications, on energy intake at a test meal after 12 weeks of oral administration of treatment.

## Secondary objectives

To evaluate the changes in the following outcome measures with dapagliflozin, 10 mg daily, after 7 days and at 12 weeks, compared with placebo, when added to up to two other oral glucose-lowering medications on:

1. Energy intake and appetite expression at a test meal after 7 days (hunger and satiety).
2. Total energy expenditure and RQ derived from ventilated hood measurements after 7 days and after 12 weeks.
3. Changes in central nervous system (CNS) responses to food images using blood oxygen level dependent (BOLD) fMRI after 7 days and after 12 weeks (reward driven eating).
4. Visceral and subcutaneous adipose tissue volumes (VAT/SAT) using MRI after 12 weeks.
5. Liver fat, using proton magnetic resonance spectroscopy (^1^H-MRS).

In addition, this study will address the effects of dapagliflozin on the rate of eating and satiety quotient.

## Methods and analysis

### Overall design, investigational plan and study population

**Figure 1:**
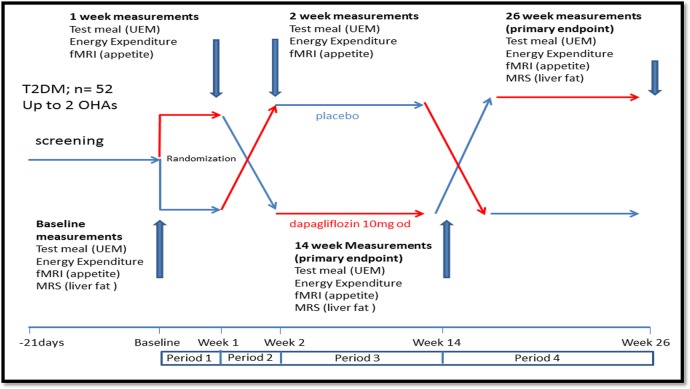
Study schematic.

**Figure 2:**
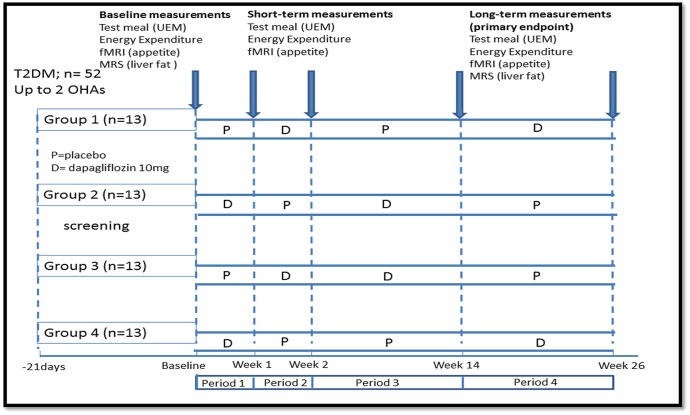
Study crossover schematic.

This will be an outpatient, double-blind, placebo-controlled crossover study, to compare the effects of dapagliflozin with placebo on food intake and energy expenditure over periods of 7 days and 12 weeks. Fifty-two participants with T2DM will be recruited: aged 18–75 years, with glycated haemoglobin (HbA1c) <11% (97 mmol/mol) and either HbA1c ≥6.5% (48 mmol/mol) in patients who are not on sulphonylureas (eg, gliclazide, glimepiride), or HbA1c ≥7.0% (53 mmol/mol) in patients who are on sulphonylureas. Each participant will visit the study centre on 12 occasions: first, to undertake routine screening tests. If identified as eligible, subsequent visits involve consuming a test meal at baseline or following placebo or dapagliflozin 10 mg, respectively, and to undertake MR measurements. All patients will receive 7 days of dapagliflozin or placebo followed by 12 weeks of each treatment. Each participant will serve as their own control with the crossover between drug and placebo at short-term and long-term. The 7-day cross-over measurements will determine the short-term effects of energy loss on food intake and energy expenditure (at this stage, it is unlikely that significant weight loss would have occurred), while the long-term crossover at 12 weeks investigates the compensatory changes at dynamic stages of weight loss. The overall study design is illustrated (figure 1), with the full crossover design shown as study schematic (figure 2). Participants will be randomised to one of the four treatment sequences (figure 2).

The inclusion and exclusion criteria for the study are summarised below.

### Inclusion criteria

1. T2DM, either treated with diet alone or up to two other oral agents (excluding pioglitazone) with an HbA1c ≥6.5% (48 mmol/mol) in patients who are not on sulphonylureas (eg, gliclazide, glimepiride) and ≥7.0% (53 mmol/mol) in those patients who are on sulphonylureas and <11% (97 mmol/mol).
2. BMI 20–50 kg/m^2^.
3. Men and women, age 18–75 years.

### Exclusion criteria

1. Medical history and concurrent diseases
  - Type 1 diabetes mellitus.
  - History of diabetic ketoacidosis or hyperosmolar non-ketotic coma.
  - Hyperthyroidism.
  - Hypothyroidism (although participants with a normal TSH and free T4, and on a stable dose of thyroxine for at least 3 months, may be included).
  - Uncontrolled hypertension (blood pressure >150/90 mm Hg).
  - Recent (<6 months) myocardial infarction.
  - Previous stroke.
  - Significant cardiac dysrhythmias (including pacemaker or implantable cardiac defibrillator device).
  - Known liver cirrhosis, viral hepatitis or autoimmune liver disease.
  - Familial renal glycosuria.
  - History of seizures or unexplained syncope.
  - Pregnancy.
  - Recent major change in body weight (>3 kg loss or gain in preceding month).
  - BMI <20 kg/m^2^.
  - History of malignancy.
  - Presence of any other medical condition that would, in the opinion of the investigator, preclude safe participation in the study.
  - Alcohol consumption in excess of daily recommended limits (14 units/week women, 21 units/week men).
  - Any history of internal metal, pacemakers or ferromagnetic metallic implants intraocular foreign bodies or cerebral aneurysm clips (exclusion from MR scanning).
2. Physical and laboratory test findings
  - ALT>3×upper limit of normal (ULN).
  - AST>3×ULN.
  - Bilirubin>2×ULN.
  - Haemoglobin≤10.5 g/dL (≤105 g/L) for men; haemoglobin≤9.5 g/dL (≤95 g/L) for women.
  - eGFR<60 mL/min.
  - Unexplained haematuria.
  - Weight>150 kg (due to limitations of MRI scanner).
3. Allergies and adverse drug reactions
  - Any history of any serious hypersensitivity reaction to dapagliflozin or SGLT-2 inhibitor.
  - Any allergy or intolerance to any of the study foods.
4. Sex and reproductive status
  - WOCBP (woman of childbearing potential) who are unwilling or unable to use an acceptable method to avoid pregnancy for the study duration plus 8 weeks.
  - Women who are pregnant or breastfeeding.
  - Sexually active fertile men not using effective birth control if their partners are WOCBP.
5. Prohibited treatments and/or therapies
  - Diabetes treated with pioglitazone, GLP-1 analogues or insulin.
  - Use of other weight loss medication or any drug that might affect body weight or appetite (including antidepressants, antipsychotics, corticosteroids).
  - Patients who are already receiving dapagliflozin or another SGLT inhibitor.
  - Patients who have participated in a SGLT2 clinical trial within the past 30 days.
  - Patients who are currently receiving a loop diuretic.
6. Other exclusion criteria
  - Prisoners or participants who are involuntarily incarcerated.
  - Participants who are compulsorily detained for treatment of either a psychiatric or physical (eg, infectious disease) illness.

### Outcome measures

The primary outcome is the change in energy intake at 12 weeks. Participants with T2DM who are recruited to similar trials typically have a mean weight of 85–95 kg and are sedentary or minimally physically active with an expected daily energy expenditure of 1780–2100 kcal. The energy loss secondary to dapagliflozin treatment is 300 kcal/day, and the placebo-subtracted weight loss is 1.5–2 kg equivalent to ∼62–83 kcal/day. Thus, the expected compensatory increase in energy intake is ∼220–240 kcal/day. Accounting for the osmotic diuresis and 10% compensatory increase as a result of this, this would further increase to ∼240–260 kcal/day (12.5–13.4% of intake).

### Sample size

The primary outcome measure is energy intake after 12 weeks. On the basis of previous research,^12^ we estimate that 52 participants are required in order to detect a change in energy intake of 12.5% with 80% power and at a two-sided 5% level of significance. This estimate is based on a correlation between measurements of 0.7 and a between-participant SD of 165, both of which are based on a previous trial.^12^ The change in energy intake of 12.5% is based on a baseline consumption of 460 g in the test meal (ie, a 12.5% change equates to a change of 57.5 g). This calculation incorporates an allowance for participant dropout of 20%. The estimate was calculated using *PROC POWER* in SAS V.9.3 and is based on the paired *t*-test.

The study is not formally powered to detect differences in liver or visceral fat, but in a previous study we were able to detect a clear reduction or 7–11% of subcutaneous and visceral adipose tissue and a reduction in liver fat of 42% (IQR: −59.3 to −16.5%) with GLP-1 analogue treatment on a study of n=25 in a pre-post treatment analysis.^11^

Recruitment will take place over 12 months and will take place in Aintree University Hospital, Liverpool, UK (four to five patients per month). Participants will be recruited either from existing databases of volunteer patients, diabetes clinics in the hospital and community and by advertisement in the local press. The randomisation for each stratum will be performed within balanced blocks to ensure approximately equal numbers of participants across the treatment sequences within each stratum. The randomisation will be performed in two strata, female and male participants.

### Screening, enrolment and randomisation

After giving written informed consent, potentially eligible participants will attend a screening visit within 6 weeks prior to randomisation; this includes a medical history to confirm the participant's eligibility to participate as determined by the inclusion/exclusion criteria, physical examination, blood tests, urinalysis and an ECG. A decision as to the participant's eligibility will be made once all the results are available. The number and size of tablets will be identical for the investigational products (dapagliflozin 10 mg and placebo) for the four treatment sequences.

### Dosage and administration of study treatments

Participants will be instructed to take a tablet with water (dapagliflozin 10 mg or matching placebo) each morning for the duration of the study (26 weeks) while continuing with their other usual medication and to attend for scheduled study visits. Participants will be asked to return all unused investigational products, including any empty packages to the study pharmacy at each visit. The participant's compliance will be discussed at each study visit and assessed based on returned tablet counts.

### Study visits and procedures

#### Test meal visits (visits 1, 3, 5, 9 and 13)

These will take place at baseline, after 7 days and 14 days, 14 weeks and 26 weeks of treatment (tables 1 and 2). On visit 1, participants will be asked to attend the investigational unit at 8:00, having had nothing to eat or drink other than water from midnight. To keep study procedures identical for all test days, participants will then be asked to take a dose of (single blind) placebo tablet. Participants will be weighed and their pulse and blood pressure recorded. An explanation and demonstration of Visual Analogue Scale (VAS) questionnaires, universal eating monitor (UEM) and ventilated hood will be given.

**Table 1:** Study procedures

Time point guidelines	Procedure
Arrival 8:30	7 mL blood sample taken (visit 9 only)
	Placebo tablet taken
	Weight, pulse and blood pressure taken. Participants asked to empty bladder and start of timed urine collection
08:40	Basal metabolic rate measurement (indirect calorimetry—see below for details)
08:50	Participants will complete a series of pre-breakfast VAS ratings to measure hunger and other subjective variables
09:00	Participants will be provided with a fixed-quantity breakfast, consisting of cornflakes with milk, toast and preserve, tea/coffee and orange juice
10:00, 11:00, 12:00 (first one followed by 3 every hour)	Participants will complete the same set of VAS ratings as used pre-breakfast, providing a premeal set of ratings
10:00	Indirect calorimetry
11:00	Indirect calorimetry
12:00	Indirect calorimetry
13:00	Participants will be given a test meal in the UEM laboratory. The meal consists of a pasta dish with tomato sauce, and participants may eat ad lib and signal when they have finished the meal. The UEM will continuously monitor decreases in food as it is consumed and will provide a continuous record of food consumption throughout the meal
14:00, 15:00, 16:00, 17:00 (1st one followed by 3 every hour)	Participants will complete the same set of VAS ratings as used pre-breakfast, providing a postmeal set of ratings
14:00	Indirect calorimetry
15:00	Indirect calorimetry
16:00	Indirect calorimetry

UEM, universal eating monitor; VAS, Visual Analogue Scale.

**Table 2:** Assessments and procedures during screening and study visits

Procedure	D −21 to −2	D 0	D 5–6	D 7	D 12–13	D 14	W 6	W 10	W 13–14	W 14	W 18	W 22	W 25–26	W 26
Visit	0	1	2	3	4	5	6	7	8	9	10	11	12	13
Informed consent	X													
Physical examination	X									X				X
Fasting visit		X		X		X				X			X	X
Bloods	X									X				X
Pregnancy test	X													
Urinalysis	X													X
Height	X													
Weight	X			X		X				X				X
BP, pulse	X			X		X								X
ECG	X													X
Glucometer provision and training	X													
Drug dispensing	X	X		X		X				X				
Drug administration				X		X	X	X	X	X	X	X	X	X
AE reporting				X		X	X	X	X	X	X	X		X
Con-med check	X			X		X	X	X	X	X	X	X		X
Compliance check				X		X	X	X	X	X	X	X		X
8 hour urine		X		X		X				X				X
Telephone reminder		X					X	X			X	X		
Test meal		X		X		X				X				X
TFEQ	X													X
fMRI	X		X		X				X				X	
MR/MRS	X								X				X	

AE, adverse event; BP, blood pressure; D, day; fMRI, functional MRI; MRS, magnetic resonance spectroscopy; TFEQ, three factor eating quotient; W, week.

This procedure will be followed on additional test days (visits 2, 6 and 10). Drug/placebo administration will take place before the fixed breakfast is consumed on each occasion.

##### Food intake and eating behaviour

Food intake and within meal assessment of hunger, fullness, desire to eat and prospective consumption will be collected using the Sussex Ingestion Pattern Monitor (SIPM V.2.0.13, University of Sussex), which comprises a concealed digital balance linked to a computer system. This system is similar to the UEM and allows continuous recording of food intake throughout a meal as well as custom programming for presentation of digital VASs pre-meal and post-meal, and at set intervals during the meal (every 150 g). Using this technology not only offers accurate collection of cumulative intake data, but also records data on subjective within meal measures of satiation. The assessment of within meal measures of satiation provides a behavioural correlate of satiety processes during a meal.^13^ Thus, the mechanisms by which potential increases in intake occur can be characterised; for example, intake may be increased due to weakened within meal satiation/delayed development of satiety. Eating rate during the meal can also be assessed using SIPM, as well as meal duration, satiety quotient and ratings of palatability, providing essential information about drug effects on the microstructure of eating. Pen and paper VAS measures of appetite (hunger, fullness, desire to eat, prospective consumption, satisfaction, thirst and nausea) fluctuations throughout the day will also be collected allowing analysis of drug effects on satiety between meals. Taken together these data fully characterise drug effects on appetite and eating behaviour. All intake, appetite and eating behaviour measurements are taken at baseline, 7 days, 14 days, 14 weeks and 26 weeks.

##### Indirect calorimetry

Energy expenditure, resting metabolic rate (RMR) and RQ will be measured by indirect calorimetry data using a ventilated hood system and derived using the Weir equation.^14^ ^15^ Measurements will be started after 5 min and performed for a 20 min period prior to the test meal and at 60, 120, 180 and 240 min postprandially. The first 5 min of each measurement will be discarded to allow for complete acclimatisation to the hood and the recumbent position, so that participants will have reclined for 10 min prior to any measurements being recorded for analysis. Urinary nitrogen excretion will be estimated by collecting all urine produced over the eight-hour period of observation in the laboratory and multiplying by a factor of 3 to approximate 24-hour urinary nitrogen excretion. Urine glucose excretion will also be measured over the same time period.

#### Imaging visits (visits 0, 2, 4, 8, 12)

Participants will be asked to attend the MRI unit on a separate day to the test meal; baseline measurements will be taken after screening and no more than 1 week before baseline test meal; other visits should take place within 1 week before or after the relevant test meal (for the week 24 visit this should be in the week preceding the final study visit/meal).

##### Neural response to food cues (fMRI)

BOLD response to food images is assessed using a 3.0 Tesla Siemens Trio scanner (Siemens, Erlangen, Germany). Participants view sets of images of high calorie (hedonic) foods, low calorie (non-hedonic) foods and non-food control objects, in a fasted state, then again 1 and 3 hours after consuming a fixed load breakfast. This is conducted to understand drug effects on the central mechanisms underpinning eating behaviour. The brain areas of interest are defined as hypothalamus, lingular gyrus, posterior insula, anterior cingulate gyrus, pre-central gyrus, posterior cingulate gyrus, middle frontal gyrus and lateral orbitofrontal cortex. Activation in areas of the brain involved in regulatory/homeostatic control of appetite (insula/hypothalamus) can be assessed on and off drug, in different states of satiety and over time. Furthermore, this design allows for observation of drug-related changes to reward neurocircuitry in response to hedonic versus non-hedonic food images. Neural pathways associated with reward-related behaviour and motivation to consume interact with homeostatic brain regions to control appetite and energy balance, thus fMRI data can provide a neurophysiological correlate of drug effects on food reward and homeostatic hunger.^16^ These measurements will be made at baseline, and at 5 or 6 days, 12 or 13 days and at 13–14 and 25–26 weeks (before the test meal evaluations). MR images will be imported into Statistical Parametric Mapping software (SPM12) for processing, followed by statistical analysis to test for significant regional BOLD response change. Following the fMRI session in the fasted state trial participants will be provided with breakfast.

##### Body composition

Participants will undergo MR whole body scanning in a 1.5 T Siemens Symphony scanner (Siemens Medical Solutions, Erlangen, Germany) at a single site, the University of Liverpool Magnetic Resonance and Image Analysis Research Centre, as previously described.

##### Liver fat by MRS and body fat volume by MRI (visit 0, 8, 12)

These measurements will be made at baseline, and at 14 and 26 weeks. Proton magnetic resonance spectroscopy of the liver will be performed as previously described.^11^ Three voxels of interest will be identified in the liver standard sites avoiding ducts and vasculature. In liver, voxel placement in post-treatment studies will be guided by reference to the pretreatment images. ^1^H MR spectra will be quantified using the AMARES algorithm in the software package jMRUI V.3.0. Intrahepatocellular lipid content (IHCL) is expressed as percentage of CH2 lipid signal amplitude relative to water signal amplitude after correcting for T1 and T2.^17^ Fat quantification by ^1^H-MRS has been validated against gold standard biochemical measurements.^18^ The mean interexamination coefficient of variation (CoV) for using this protocol is 7% (range 4–12%), and the mean intraexamination CoV is 6%.^17^

##### Changes in visceral and subcutaneous fat volume by MRI

Abdominal subcutaneous adipose tissue (abdominal SAT) and abdominal visceral adipose tissue (abdominal VAT) will be calculated from whole body axial T1-weighted fast spin echo scans (axial scans, 10 mm slice thickness followed by a 10-mm gap using the integral body coil). All images will be anonymised and blinded to time point, but not to subject (to facilitate matching anatomical landmarks), and analysed by Vardis (Vardis Group, London, UK) using SliceOMatic (Tomovision, Montreal, Canada), as described previously:^11^ the mean CoV for this methodology is total body fat, 1–2%; total subcutaneous fat, 3–4%; abdominal subcutaneous fat, 1–3%; visceral fat, 6–8%.

#### Monitoring/dispensing visits (6, 7, 10, 11)

These visits will involve a brief consultation with the study team to review glycaemic control (self-monitored capillary blood glucose), adverse effects and compliance (tablet count) and to collect supply of medication.

#### Pharmacovigilance

All adverse events will be reported and assignment of the severity/grading (mild, moderate, severe, life-threatening, death) made by the investigator responsible for the care of the participant. The assignment of causality will be made by the investigator. All non-serious adverse events (SAEs), whether expected or not, will be recorded and updated at each study visit. All new SAEs will be reported from the point of consent until 70 days after discontinuation of the investigational medical product (IMP); this includes those thought to be associated with protocol-specified procedures. Investigators will report SAEs, serious adverse reactions (SARs) and sudden unexpected adverse reactions (SUSARs) to LCTU within 24 hours of the local site becoming aware of the event. LCTU will notify the Medicines and Healthcare products Regulatory Agency (MHRA) and main REC of all SUSARs occurring during the study: fatal and life-threatening SUSARs within 7 days of notification and non-life-threatening SUSARs within 15 days. All adverse events will be followed until satisfactory resolution or until the investigator responsible for the care of the participant deems the event to be chronic or the patient to be stable.

#### Trial monitoring and oversight committees

The study will be overseen by the Trial Steering Committee (TSC). Day-to-day running of the trial will be coordinated by the Trial Management Group (TMG) supported by the LCTU, which will consist of the protocol committee members and the trial manager. The responsibilities will include

- Report to the TSC.
- Maintain the Trial Master File.
- Confirm all approvals are in place before release of the trial treatment and the start of the trial at a site.
- Provide training about the trial.
- Provide study materials.
- Data management centre.
- Give collaborators regular information about the progress of the study.
- Respond to any questions (eg, from collaborators) about the trial.
- Ensure data security and quality and observe data protection laws.
- Safety reporting.
- Ensure trial is conducted in accordance with the ICH GCP.
- Statistical analysis.
- Publication of trial results.

The role of the TSC is to provide overall supervision of the trial. In particular, the TSC will concentrate on the progress of the trial, adherence to the protocol, patient safety and consideration of new information. The TSC must be in agreement with the final protocol and, throughout the trial, will take responsibility for:

- Major decisions such as need to change the protocol for any reason.
- Monitoring and supervising the progress of the trial.
- Reviewing relevant information from other sources.
- Considering recommendations from the DMC.
- Informing and advising the TMG on all aspects of the trial.

The TSC will include experienced diabetes patients, other medical experts and clinical trialists. Meetings will be held at regular intervals determined by need, but no less than once a year. The ultimate decision for the continuation of the trial lies with the TSC.

### Outline of analysis

#### General approach

Continuous baseline characteristics will be summarised using mean, SD, median and interquartile range. Categorical variables will be summarised as frequencies and percentages. All point estimates will be considered to be statistically significant at the 5% level (two-sided) and will be presented with accompanying 95% CIs. No adjustment will be made for multiple comparisons.

#### Analysis

Primary and secondary outcome variables will be analysed using a covariance pattern linear mixed model (or other mixed model); the treatment effect will be adjusted for sex as a main effect (since it is a stratification factor), prerandomisation baseline values and the effect of period where relevant. An interaction test will be used to assess the treatment by period interaction (which may be indicative of a carry-over effect) and results interpreted cautiously if this is found to be significant at the 10% significance level. Although a common analysis strategy under such circumstances is to undertake a between-group treatment comparison using only the results from period 1, we follow the advice of Senn^19^ ^20^ in avoiding doing so because of the inflation in the type I error rate when conducting such a comparison conditional on the interaction test being statistically significant.

#### Rate of eating and satiety quotient

Cumulative intake data will initially be analysed for between-condition differences at each time point (every minute). These Dunnett's t-tests will be corrected conservatively for multiple comparisons (p<0.001). A standard technique for the examination of individual cumulative intake curves, relying on the visual judgement of deceleration by raters blinded to condition, will be used.^21^ This assessment will be quantified by raters calculating the coefficient of the start and end of individual cumulative intake curves to confirm their initial assessment. Curves whose coefficients are not lower at the end of the meal than at the start are classified as non-decelerating. The between-participant difference will be tested using a within participants χ^2^ test (McNemar test).

#### fMRI analysis

MR images will be imported into the Statistical Parametric Mapping software (SPM12) for processing, followed by statistical analysis to test for significant regional BOLD response changes.

#### Safety analysis

Information relating to adverse events (including events relating to hypoglycaemia and urinary and genital tract infections) will be tabulated and summarised descriptively. Continuous laboratory values will be summarised as described above.

## Dissemination

This study is being conducted in accordance with Good Clinical Practice (GCP), as defined by the International Conference on Harmonisation (ICH) and in compliance with the European Union Directive 2001/20/EC transposed into UK law as statutory instrument 2004 No 1031: Medicines for Human Use (Clinical Trials) Regulations 2004 and all subsequent amendments and the United States Code of Federal Regulations, Title 21, Part 50 (21CFR50). The trial protocol has received the favourable opinion of the NRES North West—Liverpool Central Research Ethics Committee (14/NW/0340; protocol number UoL000987).

An appropriate patient information sheet and consent forms describing in detail the trial interventions/products, trial procedures and risks were approved by the ethical committee (IEC), and the patient will be asked to read and review the document. The investigator will then explain the study to the patient and answer any questions that may arise. A contact point where further information about the trial may be obtained will be provided. After being given adequate time to consider the information, the patient will be asked to sign the informed consent document. A copy of the informed consent document will be given to the patient for their records and a copy placed in the medical records, with the original retained in the investigator site file. The patient may withdraw from the trial at any time by revoking the informed consent. The rights and welfare of the patients will be protected by emphasising to them that the quality of medical care will not be adversely affected if they decline to participate in this study.

## Regulatory approval

This trial has been registered with the MHRA and has been granted a Clinical Trial Authorisation (CTA). The CTA reference is its EudraCT Number: 2013-004264-60.

## Publication

The results will be analysed together and published as soon as possible. The Uniform Requirements for Manuscripts Submitted to Biomedical Journals (http://www.icmje.org/) will be respected. The ISRCTN allocated to this trial would be attached to any publications resulting from this trial.
